# The hormone response element mimic sequence of GAS5 lncRNA is sufficient to induce apoptosis in breast cancer cells

**DOI:** 10.18632/oncotarget.7173

**Published:** 2016-02-03

**Authors:** Mark R. Pickard, Gwyn T. Williams

**Affiliations:** ^1^ Apoptosis Research Group, School of Life Sciences, Keele University, Keele ST5 5BG, United Kingdom

**Keywords:** GAS5, lncRNA, apoptosis, breast cancer, oligonucleotide therapy

## Abstract

Growth arrest-specific 5 (GAS5) lncRNA promotes apoptosis, and its expression is down-regulated in breast cancer. GAS5 lncRNA is a decoy of glucocorticoid/related receptors; a stem-loop sequence constitutes the GAS5 hormone response element mimic (HREM), which is essential for the regulation of breast cancer cell apoptosis. This preclinical study aimed to determine if the GAS5 HREM sequence alone promotes the apoptosis of breast cancer cells. Nucleofection of hormone-sensitive and –insensitive breast cancer cell lines with a GAS5 HREM DNA oligonucleotide increased both basal and ultraviolet-C-induced apoptosis, and decreased culture viability and clonogenic growth, similar to GAS5 lncRNA. The HREM oligonucleotide demonstrated similar sequence specificity to the native HREM for its functional activity and had no effect on endogenous GAS5 lncRNA levels. Certain chemically modified HREM oligonucleotides, notably DNA and RNA phosphorothioates, retained pro-apoptotic. activity. Crucially the HREM oligonucleotide could overcome apoptosis resistance secondary to deficient endogenous GAS5 lncRNA levels. Thus, the GAS5 lncRNA HREM sequence alone is sufficient to induce apoptosis in breast cancer cells, including triple-negative breast cancer cells. These findings further suggest that emerging knowledge of structure/function relationships in the field of lncRNA biology can be exploited for the development of entirely novel, oligonucleotide mimic-based, cancer therapies.

## INTRODUCTION

Long non-coding RNAs (lncRNAs) are now recognized as a major component of the human transcriptome, but the vast majority of these molecules remain to be functionally annotated [1, 2]. Nonetheless, key regulatory roles in fundamental cellular processes are already evident for several well characterized lncRNAs [3 – 5], and dysregulated expression of these may contribute to the pathogenesis of major diseases, notably cancer [3, 6, 7]. Consequently, the lncRNAs may offer new targets for the development of novel diagnostics and therapeutics.

The *growth arrest-specific 5* (*GAS5*) gene is of particular relevance in this regard. *GAS5* encodes lncRNA in addition to a diverse range of other non-coding RNAs, including small nucleolar RNAs, PIWI-interacting RNAs and possibly micro RNAs (miRNAs) [8–10]. GAS5 lncRNA is down-regulated in multiple cancers [11], including breast cancer [12]. In such cancers, clinico-pathological characteristics show inverse correlations with GAS5 lncRNA levels, and low GAS5 lncRNA levels are often predictive of poor prognosis [11]. A tumour suppressor role for GAS5 lncRNA is further indicated by its inhibition of tumour growth in xenograft models of breast and other cancers [11, 13].

At the cellular level, GAS5 lncRNA promotes growth arrest and/or apoptosis of multiple cell types [11], including hormone-sensitive and –insensitive breast cancer cells [12–14], which is likely to account for its tumour suppressor function. Since the action of radiation therapy and many chemotherapeutics depends on the engagement of the apoptotic machinery [15, 16], this is likely to be significant from a therapeutic perspective. Indeed, down-regulation of GAS5 lncRNA levels attenuates apoptosis induction by a broad range of treatments; for most, cell death shows a direct quantitative relationship with GAS5 lncRNA levels in breast and other cancer cells [14, 17, 18]. Consequently, enhancing cellular GAS5 lncRNA levels in tumour tissue may not only suppress the growth of such tumours but also enhance tumour cell killing by therapeutic agents, thereby improving patient outcomes.

One way to achieve this therapeutic goal may be to target the physiological mechanism that mediates the accumulation of GAS5 lncRNA levels in growth-arrested cells. *GAS5* possesses a 5′-terminal oligopyrimidine (5′-TOP) sequence, therefore its translation is promoted by mTOR, which has high activity in actively growing cells [8, 19]. Because the *GAS5* open reading frame is short and does not encode a functional protein, this in turn targets transcripts for degradation by nonsense-mediated decay (NMD), resulting in low cellular levels of GAS5 lncRNA [8, 19]. Conversely, inhibition of cell growth and mTOR activity prevents the active translation of GAS5 transcripts. Since degradation through NMD is dependent on active translation of the RNA concerned, GAS5 lncRNA accumulates upon growth arrest [8, 19]. While mTOR inhibitors increase GAS5 lncRNA levels in hormone-sensitive breast cancer cells, they are ineffective in triple-negative breast cancer (TNBC) cells and other hormone-independent cancer cells [14, 20], so that alternative, mTOR inhibitor-independent, approaches are required to activate this key pathway across a broad range of cancer subtypes.

Several molecular mechanisms of action have been proposed for GAS5 lncRNA, which offer possibilities in this respect. Firstly, it interacts with and riborepresses certain members of the steroid nuclear receptor superfamily, and thereby modulates the transcription of genes regulating apoptosis and the cell cycle. A stem-loop structure within GAS5 lncRNA 3′-terminal sequence (exon 12 –encoded), which serves as a hormone response element mimic (HREM), is required for this interaction [21]. Secondly, it can act as a miRNA sponge, since it binds to and modulates the levels of onco-miR21; a distinct (exon 4-derived) *GAS5* sequence is required for this activity [13]. Functional analysis of mutated GAS5 lncRNA sequence has revealed that while apoptosis induction in lymphoid cells is only partially dependent on the GAS5 HREM sequence, more complete dependence is observed in breast and prostate cancer cells [22], implicating riborepression as the major mechanism by which GAS5 lncRNA induces the death of these cells. Novel oligonucleotide therapy, based on the GAS5 lncRNA HREM sequence, may therefore be feasible, but it is not yet clear if this sequence alone is sufficient to mediate apoptosis induction in epithelial cells, or if additional GAS5 lncRNA sequence is required. In order to address these questions, we have examined if the GAS5 HREM sequence alone is sufficient to promote the apoptosis of breast cancer cells, both basally and upon the application of an apoptotic stimulus, and how the resulting cellular responses compare with those of mature GAS5 lncRNA.

## RESULTS

### DNA oligonucleotides corresponding to the GAS5 HREM induce apoptosis in breast cancer cell lines

Nucleofection of a plasmid encoding mature GAS5 lncRNA induces apoptosis in hormone-sensitive and -insensitive breast cancer cell lines [12 – 14], and wild-type HREM sequence is required for this activity [22]. In initial experiments, we therefore examined if DNA oligonucleotides based on the wild-type GAS5 HREM sequence alone also induced apoptosis in a range of breast cancer cell lines; negative controls were nucleofected with DNA oligonucleotides either with stem complementarity but lacking the GAS5 HRE consensus (stem loop or SL control) or with scrambled GAS5 sequence (Scram control). Parallel nucleofection of a plasmid encoding GAS5 lncRNA (or empty plasmid vector as the respective control) was performed in some experiments, as a positive control.

**Figure 1:**
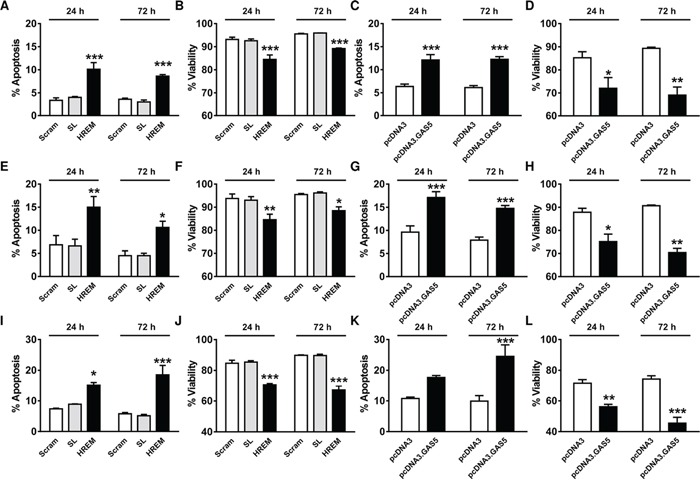
Effect of the GAS5 HREM DNA oligonucleotide on the basal survival of breast cancer cells Cells (n = 4 cultures) were transfected with the GAS5 HREM DNA oligonucleotide (HREM) or scrambled (scram) or stem loop (SL) control DNA oligonucleotides; transfections were also conducted with the plasmids pcDNA3.GAS5 (encodes mature GAS5 lncRNA) and pcDNA3 (empty control vector) as controls. Cells were harvested at 24 h *post*-transfection for assessment of cell survival, and re-plated for re-assessment after a further 48 h. The GAS5 HREM oligonucleotide increased apoptosis and decreased cell viability in MCF7 (panels **A** & **B**, respectively), T-47D (panels **E** & **F**, respectively) and MDA-MB-231 (panels **I** & **J**, respectively) cells, similar to the action of GAS5 lncRNA (panels **C** & **D**, respectively for MCF7 cells; **G** & **H**, respectively for T-47D cells; and **K** & **L**, respectively, for MDA-MB-231 cells). *P < 0.05, **P < 0.01 and ***P < 0.001 *versus* scram and SL for HREM or *versus* pcDNA3 for pcDNA3.GAS5 (two-way ANOVA and Bonferroni's MCT).

In hormone-sensitive MCF7 cells, the apoptotic rate and culture viability were similar for cells nucleofected with either the Scram or SL DNA oligonucleotides (Figure 1A & 1B); levels were indistinguishable from those of mock-transfected cells (data not shown). Nucleofection of the HREM DNA oligonucleotide approximately doubled the apoptotic rate (Figure 1A) and produced a corresponding decrease in culture viability (Figure 1B) relative to the control DNA oligonucleotides. The extent of apoptosis induction by the HREM oligonucleotide (Figure 1A) was similar to that for MCF7 cells transfected with a plasmid encoding GAS5 lncRNA (Figure 1C), whereas the latter construct produced a more sustained loss of short-term culture viability (Figure 1D) than the HREM oligonucleotides (Figure 1B). Similar results were obtained for the other hormone-sensitive cell line, T-47D, studied here. Thus, in this cell line, the HREM oligonucleotide produced an approximate doubling of the basal apoptotic rate (Figure 1E), similar to the GAS5 lncRNA plasmid construct (Figure 1G), and a corresponding reduction in culture viability (Figure 1F) which was less sustained than for the GAS5 lncRNA plasmid construct (Figure 1H).

GAS5 lncRNA has previously been shown to induce apoptosis in hormone-insensitive breast cancer cells in addition to hormone-sensitive cell lines [17, 18]. Accordingly, in the TNBC line, MDA-MB-231, the GAS5 HREM DNA oligonucleotide also increased apoptosis (Figure 1I) and reduced culture viability (Figure 1J), similar to the effects of GAS5 lncRNA (Figure 1K & 1L).

### The GAS5 HREM DNA oligonucleotide reduces the clonogenic activity of breast cancer cell lines

**Figure 2:**
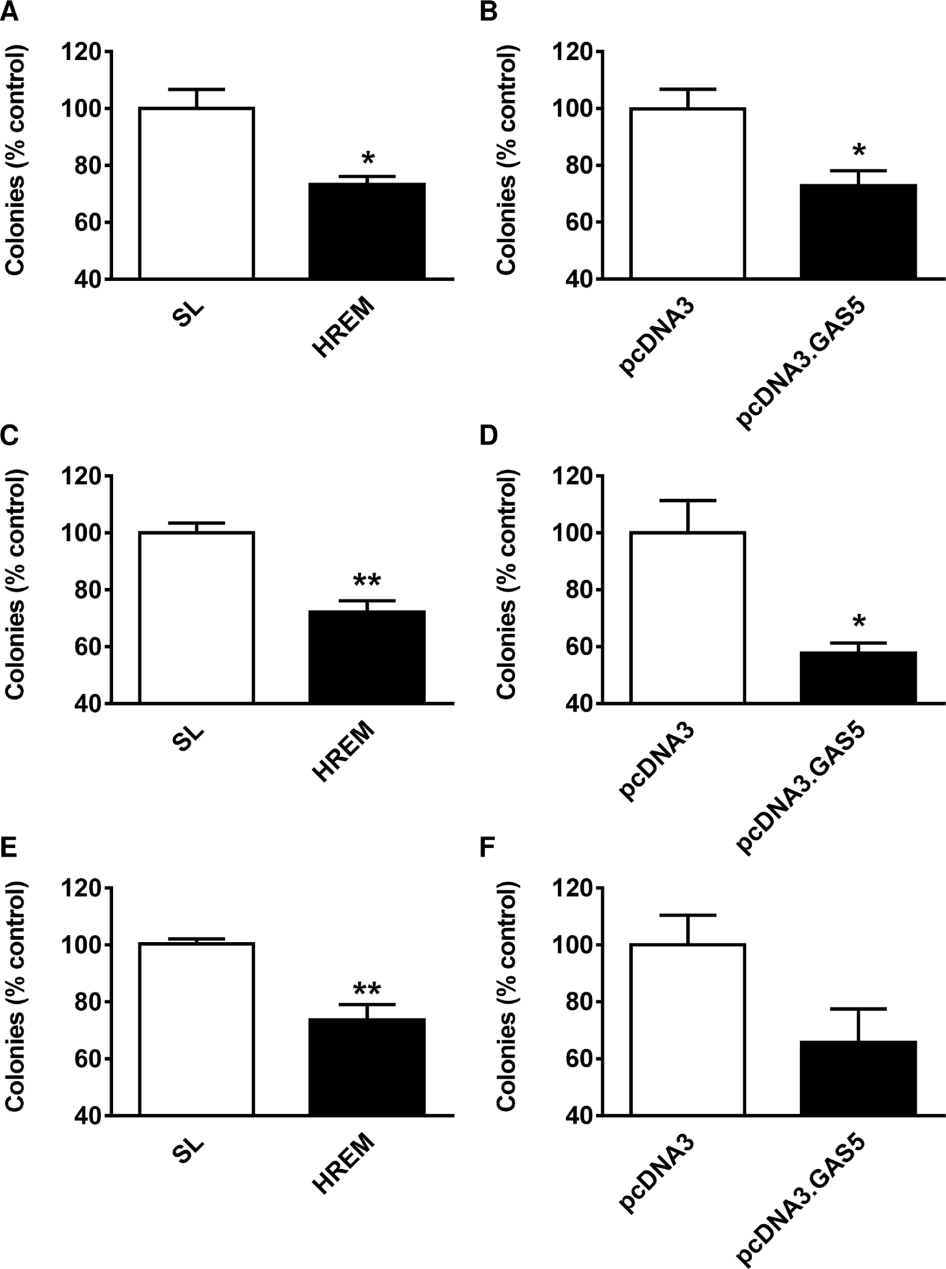
Effect of the GAS5 HREM DNA oligonucleotide on the clonogenic activity of breast cancer cells Cells (n = 4 cultures) were transfected with the GAS5 HREM DNA oligonucleotide (HREM) or scrambled (scram) or stem loop (SL) control DNA oligonucleotides; transfections were also conducted with the plasmids pcDNA3.GAS5 (encodes mature GAS5 lncRNA) and pcDNA3 (empty control vector) as positive controls. Cells were harvested at 24 h *post*-transfection and an equal portion of each re-plated at low cell density for assessment of colony forming ability; data are expressed relative to the respective control. The GAS5 HREM oligonucleotide reduced the number of colonies formed by MCF7 (panel **A**), T-47D (panel **C**) and MDA-MB-231 (panel **E**) cells, similar to the action of GAS5 lncRNA (panels **B**, **D** & **F**, respectively). *P < 0.05 and **P < 0.01 *versus* SL for HREM or *versus* pcDNA3 for pcDNA3.GAS5 (Student's *t*-test).

To determine the long-term consequences of treatment with the HREM oligonucleotide for cell survival, clonogenic growth assays were performed. Transfection of the HREM DNA oligonucleotide reduced the colony forming ability of MCF7, T-47D and MDA-MB-231 cells by 20 – 30% (Figure 2A, 2C & 2E, respectively) *i.e.*, slightly less effectively than GAS5 lncRNA (25 – 40% inhibition, Figure 2B, 2D & 2F, respectively).

### The GAS5 HREM DNA oligonucleotide promotes apoptosis induction upon DNA damage in breast cancer cell lines

**Figure 3:**
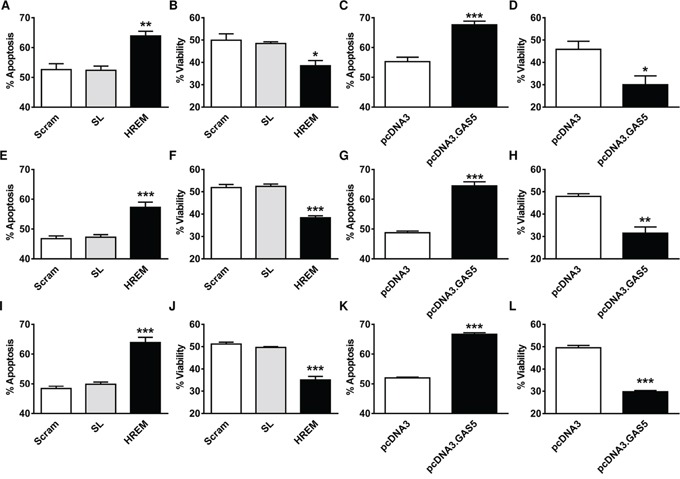
Effect of the GAS5 HREM DNA oligonucleotide on UV-C-induced cell death of breast cancer cells Cells (n = 4 cultures) were transfected with the GAS5 HREM DNA oligonucleotide (HREM) or scrambled (scram) or stem loop (SL) control DNA oligonucleotides; transfections were also conducted with the plasmids pcDNA3.GAS5 (encodes mature GAS5 lncRNA) and pcDNA3 (empty control vector) as positive controls. Cells were harvested at 24 h *post*-transfection, irradiated with UV-C light and re-plated for assessment of cell survival after a further 48 h. The GAS5 HREM oligonucleotide increased UV-C induced apoptosis and the associated loss of cell viability in MCF7 (panels **A** & **B**, respectively), T-47D (panels **E** & **F**, respectively) and MDA-MB-231 (panels **I** & **J**, respectively) cells, similar to the action of GAS5 lncRNA (panels **C** & **D**, respectively for MCF7 cells; **G** & **H**, respectively for T-47D cells; and **K** & **L**, respectively, for MDA-MB-231 cells). *P < 0.05, **P < 0.01 and ***P < 0.001 *versus* scram and SL for HREM or *versus* pcDNA3 for pcDNA3.GAS5 (one-way ANOVA and Bonferroni's MCT).

In addition to modulating the basal apoptotic rate, GAS5 lncRNA promotes apoptosis induction in breast cells by a range of treatments, including chemotherapeutic agents and physical stimuli, such as irradiation with UV-C light, which activates the DNA damage response [12 – 14]. To test whether the HREM oligonucleotide exerts similar activity to GAS5 lncRNA in this respect, breast cancer cells were nucleofected with either the HREM or control (Scram and SL) oligonucleotides and then, after 24 h, cells were irradiated with UV-C light at dosages to induce apoptosis in *ca*. 50% cells. Parallel transfections were conducted with plasmid constructs ± GAS5 lncRNA, as a positive control. Prior transfection with the HREM oligonucleotide enhanced UV-C-induced apoptosis and correspondingly decreased culture viability in MCF7 (Figure 3A & 3B, respectively), T-47D (Figures 3E & 3F, respectively) and MDA-MB-231 (Figures 3I & 3J, respectively) cells. In general, the magnitude of these effects were similar to those of GAS5 lncRNA (Figure 3C & 3D for MCF7, Figure 3G & 3H for T-47D, and Figure 3K & 3L for MDA-MB-231), further highlighting the similar activities of the GAS5 HREM and GAS5 lncRNA sequences.

### Sequence specificity of GAS5 HREM action

**Figure 4:**
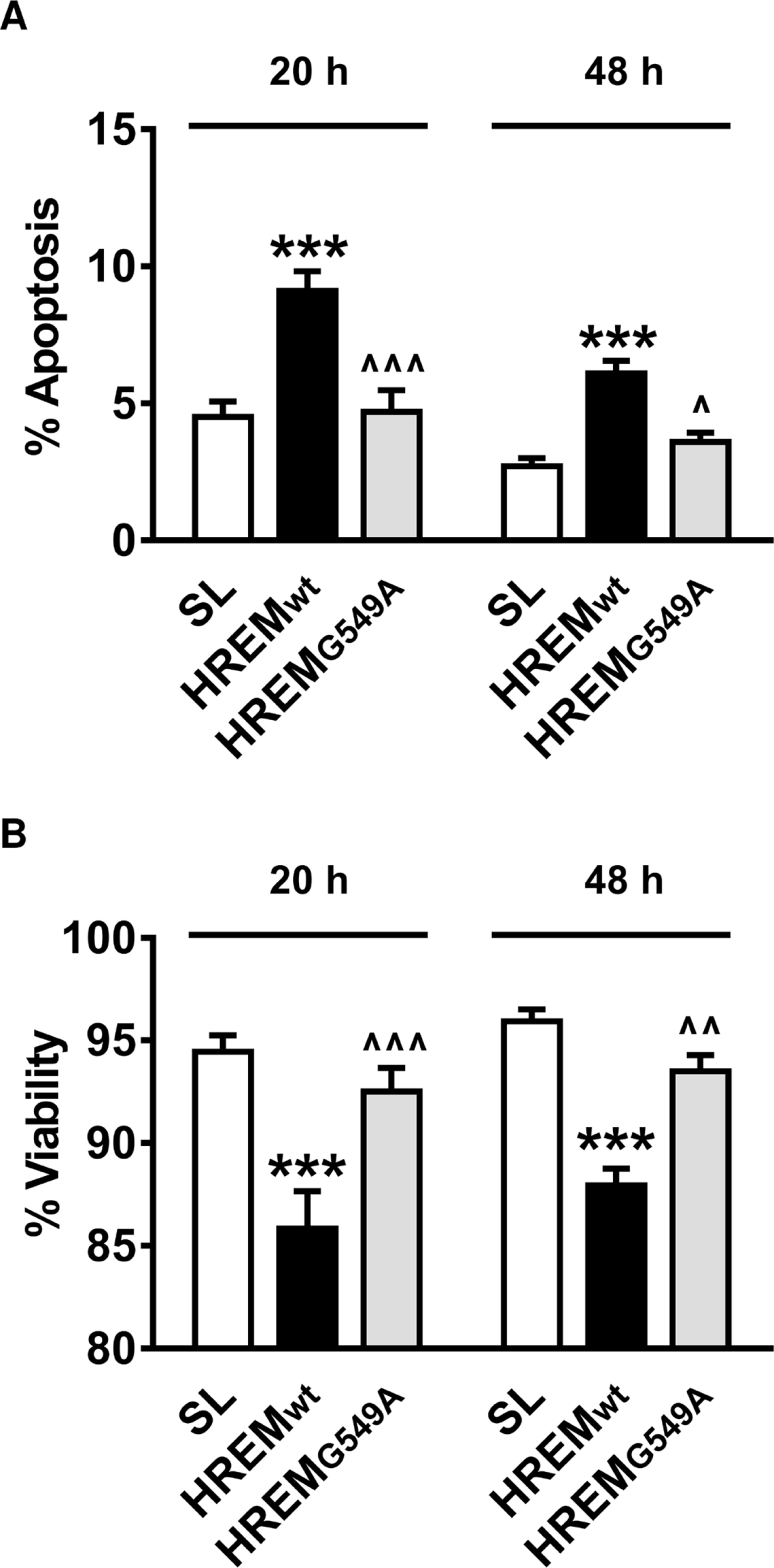
Sequence specificity of the GAS5 HREM DNA oligonucleotide MCF7 cells (n = 5 cultures) were transfected with either the wild-type GAS5 HREM DNA oligonucleotide (HREM_wt_), a mutated GAS5 HREM DNA oligonucleotide (HREM_G549A_) or the standard stem loop (SL) control DNA oligonucleotides. Cells were harvested at 20 and 48 h *post*-transfection, for assessment of cell survival. Only the HREM_wt_ oligonucleotide increased apoptosis (panel **A**) and decreased cell viability (panel **B**) relative to the SL control oligonucleotide. ***P < 0.001 *versus* SL, and ^*P* < 0.05, ^^*P* < 0.01 and ^^^*P* < 0.001 for HREM_G549A_ *versus* HREM_wt_ (two-way ANOVA and Bonferroni's MCT).

Two guanine-containing pairs (G549 and G559) within the HREM of GAS5 lncRNA are essential for its interaction with the DNA binding domain of the GR [22]. Mutation of one of these (G549A) is sufficient to inhibit the GR/GAS5 lncRNA interaction and to prevent the induction of apoptosis by GAS5 lncRNA in breast and prostate cell lines [22]. We therefore examined if induction of apoptosis by the GAS5 HREM oligonucleotide exhibited similar sequence specificity by employing a modified DNA oligonucleotide carrying the equivalent mutation. In MCF7 cells, the oligonucleotide corresponding to the wild-type GAS5 HREM sequence induced apoptosis (Figure 4A) and reduced short-term culture viability (Figure 4B) compared with the SL control oligonucleotide, whereas the effects of the oligonucleotide with mutated HREM sequence were indistinguishable from those of the SL control (Figure 4A & 4B).

### The GAS5 HREM oligonucleotide restores cell death inhibited by low endogenous GAS5 lncRNA expression

Endogenous GAS5 lncRNA levels are reduced in breast tumour *versus* adjacent normal tissue and in hormone-insensitive *versus* hormone-sensitive breast cancer cells [12 – 14]. In hormone-sensitive breast cancer cells, siRNA-mediated knockdown of GAS5 lncRNA attenuates responses to a range of pro-apoptotic treatments, including UV-C irradiation [13, 14]. To investigate if the HREM DNA oligonucleotide can overcome such resistance consequent upon low endogenous GAS5 lncRNA levels, we used siRNA to silence GAS5 expression in MCF7 cells prior to transfection of the HREM (and control) oligonucleotide, then examined cellular responses ± irradiation with UV-C light.

**Figure 5:**
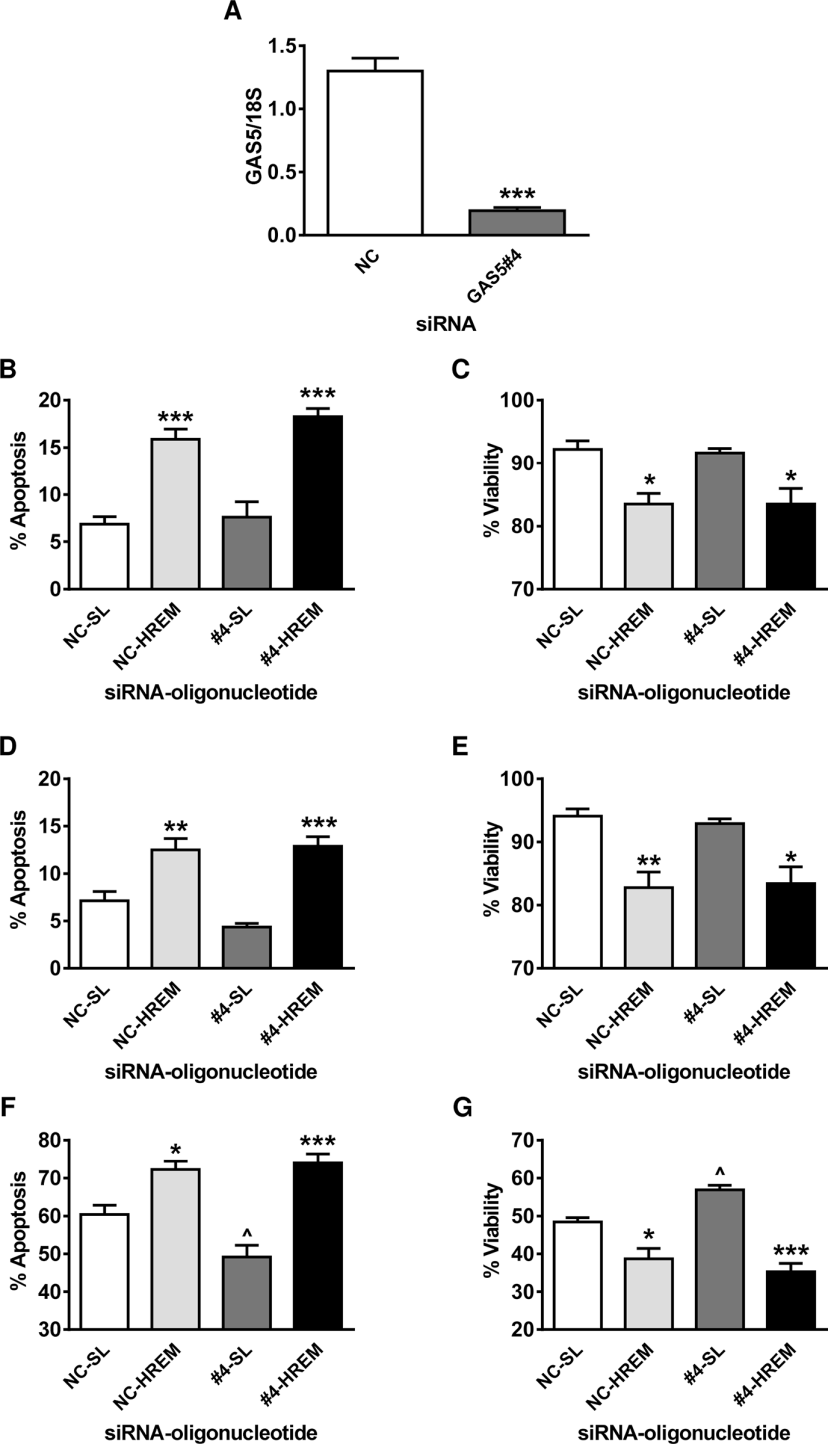
Effect of the GAS5 HREM DNA oligonucleotide on basal and UV-C-induced cell death after silencing of endogenous *GAS5* expression in MCF7 cells Cells (n = 4 cultures) were transfected with either GAS5 siRNA (GAS5#4; targets exon 12 sequence) or negative control (NC) siRNA and, after 24 h, transfected with either the GAS5 HREM DNA oligonucleotide (HREM) or stem loop (SL) control DNA oligonucleotides. After 20 h, cells were irradiated with UV-C light, then plated for assessment of cell survival after a further 48 h. The GAS5#4 siRNA markedly reduced GAS5 lncRNA levels at 24 h *post*-transfection (panel **A**) *i.e.*, immediately prior to nucleofection of DNA oligonucleotides. The HREM oligonucleotide induced apoptosis (panel **B**) and decreased culture viability (panel **C**) at 20 h *post*-transfection, irrespective of endogenous GAS5 lncRNA levels; a similar pattern was found for apoptosis (panel **D**) and culture viability (panel **E**) in mock-irradiated controls (*i.e.* at 68 h *post*-oligonucleotide transfection). Silencing of *GAS5* attenuated UV-C induced apoptosis (panel **F**) and the associated loss of culture viability (panel **G**) at 48 h *post*-irradiation, but had no effect cell death induction by the HREM oligonucleotide. Panel A: ***P < 0.001 *versus* cells transfected with NC siRNA (Student's *t*-test). Panels B – H: *P < 0.05, **P < 0.01 & ***P < 0.001 *versus* cells transfected with SL oligonucleotide; and ^*P* < 0.05 *versus* cells transfected with NC siRNA (one-way ANOVA and Bonferroni's MCT).

The GAS5 siRNA markedly reduced GAS5 lncRNA levels immediately prior to oligonucleotide nucleofection (Figure 5A) but this had no effect on basal apoptosis (Figure 5B) or cell survival (Figure 5C) at 20 h *post*-transfection of either the SL or HREM oligonucleotide; the HREM oligonucleotide produced the expected changes of similar magnitude in these parameters, irrespective of the endogenous GAS5 lncRNA levels. Cells were then treated ± UV-C light then studied after a further 48 h culture; at this time point, the mock-irradiated controls demonstrated a similar pattern of change in apoptosis (Figure 5D) and cell viability (Figure 5E) as at 20 h post-transfection (in comparison to Figure 5B & 5C, respectively), notwithstanding the expected decline in the proportion of cells undergoing active apoptosis. As regards UV-C irradiation, prior knockdown of GAS5 lncRNA clearly attenuated apoptosis induction (Figure 5F) and loss of culture viability (Figure 5G), as judged from the responses of cells which had received the SL control oligonucleotide. Crucially however, prior transfection with the HREM oligonucleotide enhanced UV-C-induced apoptosis (Figure 5F) and the associated loss of culture viability (Figure 5G), irrespective of the level of endogenous GAS5 lncRNA. Notably, the proportions of apoptotic (and viable) cells were similar for the NC-HREM and #4-HREM treatment groups, demonstrating that the HREM oligonucleotide can prevent the attenuation of cell death caused by reduced GAS5 lncRNA levels.

### The GAS5 HREM DNA oligonucleotide has no effect on endogenous GAS5 lncRNA levels

**Figure 6:**
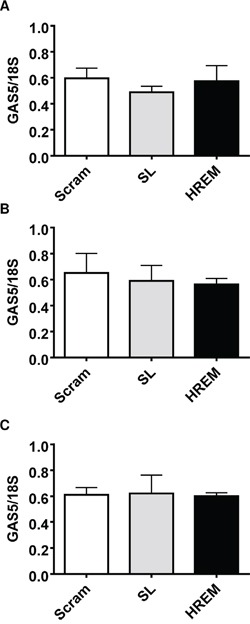
Effect of the GAS5 HREM DNA oligonucleotide on endogenous GAS5 lncRNA levels in breast cancer cells Cells (n = 4 cultures) were transfected with either the GAS5 HREM DNA oligonucleotide (HREM) or scrambled (scram) or stem loop (SL) control DNA oligonucleotides and cells were harvested at 24 h *post*-transfection for determination of GAS5 lncRNA levels by RT-qPCR. The GAS5 HREM oligonucleotide had no effect on GAS5 lncRNA levels in MCF7 (panel **A**), T-47D (panel **B**) and MDA-MB-231 (panel **C**).

GAS5 lncRNA exerts pro-apoptotic effects in breast cancer cell lines as does the GAS5 HREM DNA oligonucleotide (Figure 1A, 1E & 1I). To address the postulate that the GAS5 HREM oligonucleotide may exert its pro-apoptotic activity *via* changes in endogenous levels of GAS5, RT-qPCR analysis was performed on MCF7, T-47D and MDA-MB-231 cell lines at 20 h *post*-transfection of control and GAS5 HREM DNA oligonucleotides. In all cases, GAS5 lncRNA levels were similar between cells transfected with the GAS5 HREM and control oligonucleotides (Figure 6), excluding an indirect role for GAS5 lncRNA *per se* in GAS5 HREM oligonucleotide action.

### Chemically modified GAS5 HREM oligonucleotides also induce apoptosis

**Figure 7:**
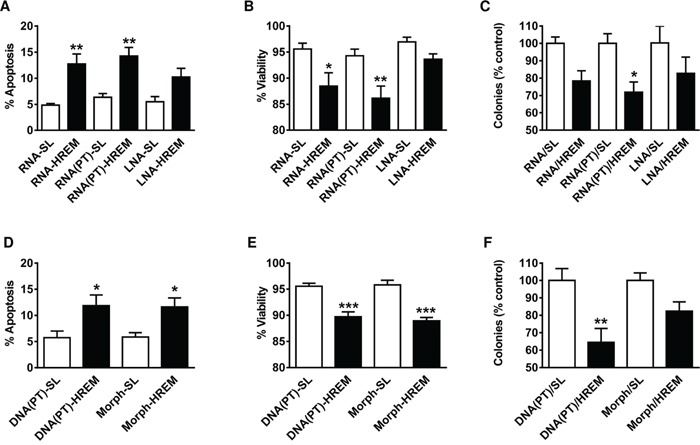
Effect of chemically modified GAS5 HREM RNA and DNA oligonucleotides on the basal survival of MCF7 cells Cells (n = 4 cultures) were transfected with a range of chemically modified GAS5 HREM and corresponding stem loop (SL) control oligonucleotides comprising: unmodified RNA (RNA-HREM & RNA-SL); phosphorothioate RNA (RNA(PT)-HREM & RNA(PT)-SL); locked nucleic acid (LNA-HREM & LNA-SL); phosphorothioate DNA (DNA(PT)-HREM & DNA(PT)-SL); and morpholinos (morph-HREM & morph-SL). Cells were harvested at 20 h *post*-transfection for assessment of cell survival, and an equal portion of each re-plated at low cell density for assessment of colony forming ability; the latter data are expressed relative to the respective control. Of the RNA-based oligonucleotides, the LNA-HREM effect did not reach statistical significance but both the RNA-HREM and the RNA(PT)-HREM oligonucleotides increased apoptosis (panel **A**) and decreased cell viability (panel **B**); the inhibition of clonogenic growth reached statistical significance for only the RNA(PT)-HREM oligonucleotide (panel **C**). Both the DNA(PT)-HREM and morph-HREM oligonucleotides also increased apoptosis (panel **D**) and decreased cell viability (panel **E**), but the inhibition of clonogenic growth reached statistical significance for only the DNA(PT)-HREM (panel **F**). *P < 0.05, **P < 0.01 and ***P < 0.001 *versus* the corresponding SL control (one-way ANOVA and Bonferroni's MCT).

Since experiments so far had employed a HREM DNA oligonucleotide, although the naturally occurring HREM is present within lncRNA, we wished to examine the efficacy of a HREM RNA oligonucleotide. Since RNA is prone to degradation, especially within exonuclease-rich cellular environments, we additionally examined several oligonucleotides with chemical modifications to the backbone that improve stability *in vivo* [23], *i.e.*, phosphorothioate RNA [RNA(PT)] and locked nucleic acid (LNA) oligonucleotides. In these screening studies, nucleofection of both unmodified RNA and RNA(PT) HREM oligonucleotides stimulated the basal apoptosis of MCF7 cells, whereas the LNA oligonucleotide was less effective and did not reach statistical significance (Figure 7A). Short-term culture viability was also reduced by the GAS5 HREM RNA and RNA(PT) oligonucleotides (Figure 7B). As regards long-term survival however, only the GAS5 HREM RNA(PT) oligonucleotide was able to reduce the colony forming ability of MCF7 cells to a degree that was statistically significant (Figure 7C).

Given these findings, it was of interest to determine if a phosphorothioate DNA [DNA(PT)] form of the GAS5 HREM also induces apoptosis in MCF7 cells. In addition, a morpholino HREM oligonucleotide was included in this screen, as the utility of this chemical modification has already been demonstrated for other types of therapeutic oligonucleotide (notably antisense oligonucleotides) [23]. Both chemically modified forms of the HREM DNA oligonucleotide induced apoptosis (Figure 7D) and reduced short-term viability (Figure 7E) in MCF7 cells. However, only the DNA(PT) oligonucleotide reduced the long-term survival of MCF7 cells to a degree that was statistically significant (Figure 7F). Notably, the extent of this reduction was similar to that of the RNA(PT) form of the GAS5 HREM oligonucleotide (compare Figure 7C & 7F).

## DISCUSSION

Currently, there is a major need to develop new targeted therapies for breast and other cancers. In the case of breast cancer, this is especially true for hormone-insensitive forms of disease, such as TNBC, which is often characterised by highly malignant behaviour and resistance to conventional chemotherapy [24]. Much effort is being focussed on the development of drugs which directly target oncoproteins [24, 25], while increasing knowledge of the importance of lncRNAs in health and disease may offer opportunities for the design of entirely novel oligonucleotide-based oncotherapies. Towards this goal, we have exploited recent knowledge regarding the structure/function relationship of the relatively well characterized GAS5 lncRNA [22], an apoptosis-promoting molecule and putative tumour suppressor [11], and demonstrate here for the first time that oligonucleotides based on the HREM sequence alone are sufficient to induce the apoptosis of breast cells. This work is of more general significance, since it provides the first proof-of-principle of using partial sequences to mimic the action of tumour suppressive lncRNA molecules and, as such, points to an innovative approach for oligonucleotide-based oncotherapies of potential clinical importance.

GAS5 lncRNA levels are down-regulated in a wide range of tumours, including breast cancer; in many cancers low levels of expression are prognostic of poor patient survival [11]. The basis for this tumour suppressor function has been ascribed to the apoptosis-promoting activity of GAS5 lncRNA, which has itself been shown to reside within the HREM portion of the GAS5 lncRNA molecule in breast and other cells [21, 22]. The present study, which employs independent assays of apoptotic morphology, short-term culture viability and long-term clonogenic survival, clearly demonstrates that the HREM portion alone of the GAS5 lncRNA molecule induces cell death in three different breast cancer cell lines, including both hormone-sensitive and –insensitive breast cancer cells. Notably, for all cells, the extent of apoptosis induction and inhibition of growth were similar for both the GAS5 HREM oligonucleotide and GAS5 lncRNA.

The GAS5 HREM oligonucleotide also enhanced the action of an external apoptotic stimulus, UV-C irradiation. For both the partial and full length GAS5 lncRNA sequences, this effect was additive rather than synergistic, as previously noted for the latter sequence [12, 14]. Of direct clinical relevance, reduced endogenous GAS5 lncRNA expression, such as occurs in breast cancer and many other tumours [11 – 14], attenuates cellular responses to apoptotic inducers, including UV-C and various chemotherapeutic agents [13, 14]; the GAS5 HREM was able to restore breast cancer cell sensitivity, as demonstrated here for UV-C irradiation. Thus in cancers with deficient *GAS5* expression, the GAS5 HREM oligonucleotide may have therapeutic potential, not only by acting as an apoptotic inducer *per se*, but also by restoring the effectiveness of more conventional oncotherapies on therapy-resistant cancer cells.

An important question relates to the mechanism of action of the GAS5 HREM oligonucleotide in promoting the apoptosis of breast cancer cells. Our current working hypothesis is that the partial HREM sequence acts as a decoy for members of the steroid hormone nuclear receptor superfamily, as has been demonstrated for GAS5 lncRNA [21, 22]. In order to test this hypothesis we examined the effect of a potentially inactivating mutation in the HREM on its cytotoxic effects. These experiments demonstrate that the HREM oligonucleotide has identical sequence specificity to GAS5 lncRNA. Thus, mutation of a single base (G549A) of the HREM of GAS5 lncRNA diminishes steroid receptor binding and ablates apoptosis induction in breast cancer cells [22], and an identical mutation of the GAS5 HREM oligonucleotide also prevents its induction of apoptosis in MCF7 cells (Figure 4), indicating that the mechanism of induction of apoptosis in breast cancer cells by the HREM is the same as that of GAS5 lncRNA. It is as yet unknown which particular member(s) of the steroid hormone nuclear receptor superfamily interact with GAS5 lncRNA in breast cells, particularly in relation to apoptosis induction; consequently, we have been unable to address this issue for the GAS5 HREM oligonucleotide. In this regard, the GAS5 HREM does not interact with estrogen receptors [21, 22], which are crucial for mediating pro-survival signalling by estrogen in breast cells [26]. While the GAS5 HREM interacts with progesterone receptor [21, 22], this interaction is unlikely to play a major role in breast cancer cell survival, since both GAS5 lncRNA [14] and the GAS5 HREM sequence induce apoptosis in TNBC cells which are devoid of progesterone receptors. On the other hand, a more indirect mechanism of action for the GAS5 HREM oligonucleotide, centring on endogenous GAS5 lncRNA *per se*, can be excluded, since: i. cellular levels of the latter were unperturbed following transfection of the GAS5 HREM oligonucleotide; and ii. siRNA-mediated silencing of GAS5 lncRNA had no effect on the apoptosis-inducing activity of the GAS5 HREM oligonucleotide in MCF7 cells.

These findings prove that for GAS5 lncRNA, it is feasible to employ a partial sequence to mimic a desirable function of the parental lncRNA and thereby point to the possibility of novel oligonucleotide mimic-based oncotherapeutic approaches. With this in mind, a range of commonly used chemical modifications which are known to improve oligonucleotide stability *in vivo* [23], were tested for the HREM oligonucleotide in functional assays. Following nucleofection, most modified HREM oligonucleotides were able to induce apoptosis and decrease short-term culture viability, although this effect was not statistically significant for the locked nucleic acid form. In terms of inhibition of long-term cell survival, phosphorothioate forms of RNA and DNA oligonucleotides appeared optimal. These findings indicate that certain, but not all, modifications to the oligonucleotide backbone are well tolerated in functional terms by the GAS5 HREM oligonucleotide.

Mimic oligonucleotides may have widespread applicability as our understanding of the structure/function relationships of lncRNA molecules increases. A particular advantage of such therapies is the ability to mimic the activity of tumour suppressor genes, which are often down-regulated in tumours, whereas current protein-targeted, drug development approaches are biased towards the inhibition of oncogenic proteins, which are typically overexpressed in tumours. Furthermore, the effectiveness of partial sequences, as seen above, makes oligonucleotide-based approaches more amenable, since these are inherently less complex and therefore more practical clinically than gene therapy approaches. Examples of other therapeutic approaches that exploit emerging knowledge of lncRNA biology and are currently under development include siRNA, other RNAi and antisense oligonucleotide approaches for targeting oncogenic lncRNAs, antagoNAT antisense oligonucleotides for targeting antisense lncRNAs to up-regulate specific mRNAs/proteins, and small molecule inhibitor (including oligonucleotides) approaches [23, 27–29]; the oligonucleotide mimic approach proposed here is likely to be compatible with and complementary to these. Indeed, the challenges for clinical translation, in terms of *in vivo* stability, tumour delivery and cellular uptake, for example, are likely to be similar for all these technologies and are currently the focus of active investigation.

## MATERIALS AND METHODS

### Cell lines and culture conditions

This study employed hormone-sensitive (MCF7 and T-47D) and –insensitive (MDA-MB-231; TNBC cells) breast cancer cell lines. All cultures were generated from secondary stocks of cells which had been frozen down within two weeks receipt from ATCC-LGC Promochem (Teddington, Middlesex, UK), and cultures were passaged for a maximum period of eight weeks before replacement with fresh cell stocks. Culture medium was R-10 medium, comprising RPMI-1640 supplemented with 2 mM L-glutamine, 1 mM sodium pyruvate, 10 mM HEPES, 10% fetal bovine serum and 50 μg/ml gentamicin, and cells were cultured at 37°C in a humidified incubator with 5% CO_2_. Serum was from Labtech International Ltd (Uckfield, UK) and the remaining cell culture reagents were from Sigma-Aldrich Company Ltd (Gillingham, UK).

### Nucleofection of oligonucleotides and plasmid DNA

DNA oligonucleotides (Eurofins Genomics, Ebersberg, Germany) were studied in the majority of experiments and comprised the wild-type GAS5 HREM sequence (5′-CAGTGGTCTTTGTAGACTGCCTG-3′), a mutated GAS5 HREM (5′-CAGTAGTCTTTGTAGACTGCCTG-3′), a control sequence which retains stem complementarity but lacks the GAS5 HRE consensus (stem loop control; 5′-CTGATGGTCTTTGTAGACCATCA-3′) and scrambled sequence (5′-TGTTGGCTTGTCACGCATGCGTCT-3′). Equivalent stem loop control and wild-type GAS5 HREM sequences were also employed in experiments with RNA oligonucleotides (Eurofins Genomics, Ebersberg, Germany), DNA and RNA phosporothioate-modified oligonucleotides (GE Healthcare Dharmacon Inc, Little Chalfont, UK) and morpholino oligonucleotides (Gene Tools LLC, Philomath, USA). Sequences of locked nucleic acid oligonucleotides (Exiqon, Vedbaek, Denmark) containing selective phosphorothioate backbone modifications (as indicated by *) were: 5′-C+T+G+A+T+G*G*T*C*T*T*T*G*T+A+G+A+C+C*A*T*C*A-3′ for the stem loop control; and C+A+G+T+G+GT*C*T*T*T*G*T*A*G+A+C+T+G+C*C*T*G-3′ for the GAS5 HREM. Cells (2 × 10^6^ in 0.1 ml Ingenio electroporation solution [Mirus Bio LLC, Madison, WI, USA]) were nucleofected with 5 pmol oligonucleotide or 2 μg plasmid (either pcDNA3/GAS5—encodes mature GAS5 lncRNA—or empty pcDNA3 vector as control; positive control experiments) using programmes E-014, X-005 and X-013 for MCF7, T-47D and MDA-MB-231 cells, respectively, and cells were plated in 3 ml R-10 medium in 6-well plates.

### RNA interference by siRNA

MCF7 cells were transfected with Ambion Silencer Select GAS5 siRNA (product code n272331; targets exon 12; Life Technologies, Paisley, UK) or negative control (NC) siRNA (product code AM4611) using HiPerFect reagent (Qiagen, Crawley, UK) and a fast forward protocol. This particular GAS5 siRNA (termed GAS5 siRNA #4) has previously been shown to silence GAS5 expression in MCF7 cells using an RNAiFect protocol [14]. Briefly, trypsinized cells (2 × 10^5^) were seeded in 2.3 ml R-10 medium per well of a 6-well plate. Complexes were prepared by mixing 12 pmol siRNA in 0.1 ml Opti-MEM-I medium with 12 μl HiPerFect reagent and, after 15 min, these were added to cells. Cells were then cultured for 20 – 24 h prior to nucleofection with the indicated oligonucleotides.

### Induction of cell death and cell survival assays

At 20 h *post*-nucleofection with oligonucleotides, cells were trypsinized and resuspended at 2 × 10^5^ cells/ml in R-10 medium. A sample was taken for RNA isolation, and cells were routinely seeded at 1.6 × 10^5^ cells/well of a 12-well plate. In experiments employing ultraviolet-C (UV-C) irradiation for apoptosis induction, this was performed immediately prior to plating, as described elsewhere [17]; the dose used was 40 J/m^2^ for all cell lines; controls were mock-irradiated. Cells were cultured for a further 48 h, then adherent cells were trypsinized and combined with non-adherent cells for analysis of parameters of cell survival.

Apoptosis was routinely determined by assessment of nuclear morphology of cells by fluorescence microscopy, after staining with acridine orange (25 μg/ml); cells containing condensed or fragmented chromatin were scored as apoptotic. Cell viability was determined by dye-exclusion assay by counting of nigrosin blue (0.1% (w/v)) stained samples using a haemocytometer and light microscopy. To determine long-term survival of cells, a clonogenic assay was performed: cells were replated in R-10 medium supplemented with 10 % (v/v) cell-conditioned medium in 6-well plates, cultured for 3 weeks and the number of colonies formed was determined *post*-staining with crystal violet.

### Real time RT-PCR (RT-qPCR)

Total RNA was isolated with TRIzol, DNase-treated, then reverse transcribed by random hexamer priming using SuperScript II Reverse Transcriptase [14]. Real-time PCR was conducted on the resulting cDNA using a SensiFast Probe Hi-ROX kit (Bioline, London, IK) and TaqMan Gene Expression Assays (assay codes Hs99999901_m1 for 18S and Hs03464472_m1 for GAS5; Life Technologies, Paisley, UK); assays usually contained 10 ng sample cDNA in a final volume of 25 μl and were run on an ABI Prism Sequence Detection System model 7000. Standards (0.2 – 60 ng cDNA from untransfected MCF7 cells) were included with each assay; the resulting standard curves of threshold cycle (C_T_) value versus log input standard cDNA were used to calculate input amounts of samples from their respective C_T_ values, and data were expressed relative to 18S rRNA.

### Statistical analyses

Data are presented as the mean ± SEM; the number of observations (n) refers to different transfected samples, each from a separate culture of cells. Data analysis was either by an unpaired Student's *t*-test (when comparing two groups only), by one-way analysis of variance, with Bonferroni's multiple comparison test (MCT), or by two-way analysis of variance, with Bonferroni's *post* tests, as indicated in the text. Homogeneity of variance was checked by Bartlett's test and, where necessary, data were transformed (log or square root) prior to analysis. Statistical analyses were performed using GraphPad Prism v4.03.
